# Synthesis and Characterization of Porous Hydrophobic and Hydrophilic Silica Microcapsules for Applications in Agriculture

**DOI:** 10.3390/ma17184621

**Published:** 2024-09-20

**Authors:** Yeela Elbaz, Taly Iline-Vul, Aviv Dombrovsky, Ayelet Caspi, Shlomo Margel

**Affiliations:** 1 Bar-Ilan Institute of Nanotechnology and Advanced Materials (BINA), Department of Chemistry, Bar-Ilan University, Ramat-Gan 5290002, Israel; yeelailuz@gmail.com (Y.E.);; 2 Department of Plant Pathology and Weed Research, Agricultural Research Organization, The Volcani Center, Rishon LeZion 7528809, Israel; 3 TAMA Group, Kibbutz Mishmar HaEmek 1923600, Israel

**Keywords:** silica microcapsules, thymol, hydrogen peroxide, thin coating, hay protection

## Abstract

Silica (SiO_2_) particles are widely used in various industries due to their chemical inertness, thermal stability, and wear resistance. The present study describes the preparation and potential use of porous hydrophobic and hydrophilic SiO_2_ microcapsules (MCs) of a narrow size distribution. First, various layers of SiO_2_ micro/nano-particles (M/NPs) were grafted onto monodispersed polystyrene (PS) microspheres of a narrow size distribution. Hydrophobic and hydrophilic sintered SiO_2_ MCs were then prepared by removing the core PS from the PS/SiO_2_ core–shell microspheres by burning off under normal atmospheric conditions or organic solvent dissolution, respectively. We examined how the size and quantity of the SiO_2_ M/NPs influence the MC’s properties. Additionally, we utilized two forms of hollow SiO_2_ MC for different applications; one form was incorporated into polymer films, and the other was free-floating. The hydrophobic microcapsules filled with 6% hydrogen peroxide were effective in killing the tomato brown rugose fruit virus (ToBRFV). The hydrophilic microcapsules filled with thymol and thin coated onto polypropylene films were successfully used to prevent mold formation for hay protection.

## 1. Introduction

Silica, or silicon dioxide (SiO₂), is one of the most abundant materials on earth, found naturally in various forms such as quartz, sand, and diatomaceous earth. Its versatility and unique properties have established it as a cornerstone in numerous applications across diverse industries [1]. The Stöber process, a method of silane monomer polymerization developed by Werner Stöber in 1968, revolutionized the synthesis of monodispersed SiO₂ particles [2]. This sol-gel technique involves the hydrolysis and condensation of silane alkoxides in a mixture of alcohol, water, and ammonia, allowing precise control over particle size, size distribution, and morphology [3]. The Stöber process has become a fundamental approach to creating advanced silica-based materials, including the hollow SiO₂ microspheres described in the present study. Recent advancements in materials science have led to significant developments in silica-based structures, particularly hollow silica microspheres (SiO_2_ MCs) [4,5]. These microcapsules, composed of sintered SiO₂ micro/nano-particles (M/NPs), have attracted considerable attention across various industries due to their unique properties and versatile applications. The exceptional characteristics of SiO_2_ MCs, including enhanced stability, controlled encapsulation capabilities, and biocompatibility [4], make them ideal candidates for a wide range of uses.

The method of creating SiO_2_ MCs involves coating polystyrene (PS) template microspheres of a narrow size distribution formed by dispersion polymerization of styrene with SiO_2_ M/NPs, followed by PS core removal [4,5,6,7]. This technique offers a scalable and reproducible approach for producing SiO_2_ MCs of a narrow size distribution, opening new avenues for encapsulating and delivering active compounds and presenting novel solutions to longstanding challenges in various fields.

One particularly promising application of these silica-based structures is the encapsulation of liquid oils and active ingredients [4,5]. This approach offers benefits such as enhanced stability, precise encapsulation, and long-term stability of the encapsulated compounds.

In the agricultural sector, these innovative materials offer potential solutions to pressing challenges facing crop production today [8]. Among these challenges, the loss of crops due to plant pathogens poses significant threats to global food security and economic stability. Viruses, bacteria, fungi, and nematodes cause diseases that lead to substantial crop yield losses, diminished food quality, and increased production costs [9].

The tomato brown rugose fruit virus (ToBRFV) poses a significant threat to tomato production worldwide [10]. While ToBRFV primarily affects tomatoes, it can also damage tobacco plants. In tobacco, the virus induces symptoms such as leaf mottling, chlorosis, and necrotic lesions [11]. These symptoms can significantly reduce the quality of tobacco leaves, impacting their commercial value. ToBRFV’s ability to infect tobacco plants further complicates control efforts, as tobacco can serve as a reservoir for the virus, facilitating its spread to nearby tomato crops.

Mold damage represents another significant challenge in crop production, particularly in hay and other stored forage crops. Mold growth in hay can occur when moisture levels exceed 14–15%, often due to improper drying or storage conditions. The presence of mold not only reduces the hay’s nutritional value but can also produce mycotoxins, which are harmful to livestock [12]. Common molds in hay include *Aspergillus*, *Penicillium*, and *Fusarium* species. These molds can cause respiratory issues in animals and humans handling the contaminated hay [13,14]. In severe cases, mold infestation can lead to spontaneous combustion of hay bales, posing a significant fire hazard. The economic impact of mold damage in hay production includes reduced feed quality, potential health issues in livestock, and the need for replacement feed.

In the realm of green pest control, hydrogen peroxide (HP, H_2_O_2_) and thymol have gained attention as environmentally friendly alternatives to conventional pesticides. HP, in liquid or vapor states, is a powerful oxidizing agent that can effectively control various plant pathogens, including bacteria, fungi, and viruses [15]. When used in appropriate concentrations, it breaks down into water and oxygen, leaving no harmful residues. Thymol, a natural monoterpenoid phenol derived from thyme oil, exhibits strong antimicrobial properties. It has shown efficacy against a wide range of plant pathogens and pests, including fungi, bacteria, and insects [16]. The combination of HP and thymol can provide synergistic effects, enhancing their overall efficacy in pest and disease control [17,18]. These compounds offer promising alternatives for sustainable agriculture, addressing concerns about pesticide resistance, environmental impact, and food safety associated with conventional chemical pesticides.

Recent studies conducted and published by our laboratory have shown promising results regarding the biocompatibility and stability of oil-filled SiO_2_ MCs [4,5]. These MCs were nontoxic to the human keratinocyte HaCaT cell line, indicating their potential for biomedical applications [4].

The present study presents novel methods for synthesizing both hydrophobic and hydrophilic SiO_2_ MCs with controlled surface properties and release kinetics. This study presents a simple and effective technique for producing these MCs using the Stöber process via polymerization of Si(OEt)_4_ onto core PS microspheres under various conditions, followed by PS core dissolution. This process allows precise control over the particle size, morphology, and surface characteristics. The advantages of this process include the ability to contain and deliver various active compounds, such as hydrogen peroxide and thymol, with tailored release profiles, inexpensive processes, and a non-polluting nature. This article explores new applications of the formed SiO_2_ MCs in green agriculture, focusing on their potential as antiviral agents against the ToBRFV in tobacco plants and as anti-mold agents for hay preservation. In addition, we present a new approach to incorporate these SiO_2_ MCs onto polymer films to create functional, durable, thin coatings. This study demonstrates the effectiveness of hydrogen peroxide-filled SiO_2_ MCs in combating ToBRFV and the effectiveness of thymol-filled SiO_2_ MCs in preventing mold growth on hay, offering promising solutions to critical challenges in crop protection and storage [17,18,19,20].

## 2. Materials and Methods

### 2.1. Materials

The following analytical-grade chemicals were purchased from Sigma Aldrich (St. Louis, MO, USA) and used without further purification: benzoyl peroxide (BP, 98%), polyvinylpyrrolidone (PVP, mw 360,000 Da), ethanol (HPLC), 2-methoxy ethanol (HPLC), ammonium hydroxide (NH_4_OH, 28%), tetraethyl orthosilicate (TEOS, 99%), styrene (99%), and thymol (98%). Hydrogen peroxide (HP, 30–32%) was purchased from Fisher Chemicals (Pittsburgh, PA, USA), and N,N′-bis (3-trimethoxysilylpropyl) urea (NN’BTMSPU) was purchased from Gelest Inc., Morrisville, PA, USA. Double distilled water was obtained from a TREION™ purification system. Tobacco plants were kindly provided by the Volcani Institute, Rishon LeZion, Israel, from the laboratory of Dr. Aviv Dombrovsky. Non-treated and corona-treated polypropylene (PP) films were provided by TAMA Ltd., Kibbutz Mishmar HaEmek, Israel.

### 2.2. Methods

#### 2.2.1. Synthesis of Polystyrene/Silica Core–Shell Microcapsules

Our laboratory has previously reported the synthesis of polystyrene/silica (PS/SiO_2_) core–shell MCs, building on our established method for producing microparticles from monodisperse polystyrene (PS). The process begins with the preparation of PS core particles of a narrow size distribution using a dispersion polymerization process of styrene, as detailed in our previous publications [6,7]. This method yields PS particles of a narrow size distribution, typically 2.7 ± 0.15 μm in diameter, as confirmed by scanning electron microscope (SEM) analysis.

The core–shell structure was then achieved through a multistep SiO_2_ coating process. The PS template particles were first dispersed in ethanol, followed by the addition of water, a basic catalyst (NH_4_OH or NaOH), and tetraethyl orthosilicate (TEOS). This initiates the formation of the first SiO_2_ micro/nano-particle layer. Subsequent layers were added using repeated cycles of TEOS addition and reaction, with the number of cycles ranging from 2 to 10 depending on the desired shell thickness.

#### 2.2.2. Synthesis of Hydrophobic Porous Hollow Silica Microcapsules of Narrow Size Distribution

After centrifugation, the PS/SiO_2_ core–shell microparticles were freeze-dried. Hydrophobic porous hollow SiO_2_ MCs with a narrow size distribution were then prepared by incubating the dry PS/SiO_2_ M/NPs in an oven at 550 °C for 8 h under normal atmospheric conditions. During this process, the PS core particles combusted to obtain uniformly hydrophobic hollow porous SiO_2_ MCs.

#### 2.2.3. Synthesis of Hydrophilic Porous Hollow Silica Microcapsules of Narrow Size Distribution

Hydrophilic SiO_2_ MCs were synthesized with a focus on preserving the hydroxyl groups present in the SiO_2_ shell of the PS/SiO_2_ core–shell particles, which are essential for various applications, especially when adhering the particles to different surfaces or dispersing in an aqueous continuous phase. Instead of using combustion to remove the PS core and induce a compression reaction between the silanol groups to form siloxane, an alternative approach was employed.

For this purpose, dry PS/SiO_2_ core–shell MCs were washed by extensive centrifugation cycles with acetone to dissolve the PS core. After centrifugation at 7500 rpm for 8 min, the obtained hydrophilic hollow SiO_2_ MCs were washed with ethanol and then dried to obtain a powder at room temperature.

During the washing steps, Fourier transform infrared (FTIR) spectroscopy samples were periodically collected to monitor the release of polystyrene, and corresponding graphs were generated. This analytical approach allowed us to investigate the transformation of hydrophobic particles into hydrophilic ones, providing valuable insights into their structure and potential applications.

#### 2.2.4. Preparation of Liquid-Filled SiO_2_ Microcapsules

To encapsulate the desired liquids within the hollow SiO_2_ MCs, dry hollow hydrophilic or hydrophobic SiO_2_ MC powder was placed in a vial, and the desired liquid was introduced dropwise through a septum into the vial while maintaining a vacuum. During the liquid addition, the SiO_2_ MC powder surface remained dry until the encapsulation process was complete, stopping when the surface became a bit wet.

Briefly, for the encapsulation process of thymol, 10 mg of the SiO_2_ MC powder was combined with 60 μL of liquid thymol. The thymol was liquefied by heating to 60 °C while the empty dry SiO_2_ microcapsules powder was maintained at 40 °C and loaded dropwise with the thymol under vacuum [21].

A similar process was performed at room temperature to encapsulate a 6 w% aqueous hydrogen peroxide (HP) solution within the SiO_2_ MCs.

#### 2.2.5. Oil-Filled Hydrophilic Silica Coatings onto Polypropylene Films

PP is a nonpolar, low surface energy material, and its hydrophobic characteristics cause poor wettability. Corona treatment is an established method in the industry for surface oxidation to achieve surface functional groups such as hydroxyls, carboxylates, and ketones, as well as higher surface polarity [20]. Polar groups increase the polar part of the surface energy and, thus, also the overall surface energy and wettability.

**Figure 1:**
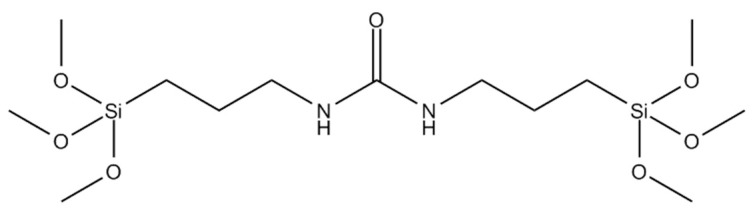
Chemical structure of N,N′-Bis(3-Trimethoxysilylpropyl)urea (NN’BTMSPU).

Oil-filled SiO_2_ MC coatings onto corona-treated PP films were prepared by using a modified Stöber polymerization method involving the monomeric bipodal silane compound N,N′-bis (3-trimethoxysilylpropyl) urea (NN’BTMSPU, see Figure 1 below), and thymol-filled SiO_2_ MCs. The EtOH/H_2_O solution containing NTMSPU and the SiO_2_ MC containing thymol were shaken at room temperature for 2–3 min and then spread onto the oxidized corona-treated PP using a Mayer rod 6 [20,22].

The composition of the solution used for coating onto corona-treated PP film was as follows:

A total of 18.75 mL EtOH, 3 mL H_2_O, 0.675 mL NH_4_OH (28%), 1.5 mL NN’BTMSPU, and 2.67 g thymol-filled SiO_2_ MC. The weight ratio between the SiO_2_ MCs and the entrapped thymol (1:3.5) was measured using a UV spectrophotometer (see Section 2.2.4 and Section 2.2.7). This method allowed for the creation of durable coatings containing oil-filled SiO_2_ MCs, providing a unique and controlled encapsulation of thymol within the material.

#### 2.2.6. Determination of Hydrogen Peroxide Concentration by KMnO_4_ Titration

The cumulative release rate of HP from SiO_2_ MCs (130 ± 2.8 nm) through vapor diffusion was investigated. For each series, SiO_2_ MCs with different numbers of coating layers (2, 6, and 10) were taken. Each series was filled with an aqueous solution of HP (6% wt) until reaching the containment capacity, which was influenced by the number of coating layers, as described in Section 2.2.4.

For the kinetics tests, 21 samples of HP-filled SiO₂ MCs, each weighing 100 mg, were placed in sealed Petri dishes with a diameter of 50 mm. The release rate of HP vapor was evaluated at specific time intervals (0, 1, 8, 24, 48, 96, and 168 h). To determine the concentration of HP released into the surrounding water, each sample was transferred to a vial containing 5 mL of double-distilled water (DDW) and treated with 325 μL of 96% sulfuric acid (H₂SO₄). The HP concentration was measured using a pre-calibrated potassium permanganate (KMnO₄) solution in a titration process. The amount of released HP vapors at each time interval was calculated as the difference in HP concentrations between time 0 and a specific time point [17,23]. The release rate tests were conducted in triplicate.

#### 2.2.7. Determination of Thymol Release Rate and Content in the Thymol-Filled SiO_2_ Microcapsules

The content of thymol in the SiO_2_ MCs was determined by a spectrophotometer [17]. The content of the thymol vapor released from the MCs was calculated by the reduction of the thymol content in the SiO_2_ MCs at time 0 from the content remaining in the MCs at different time periods. Briefly, the thymol-filled SiO_2_ MCs (100 mg) were exposed at RT for 0, 8, 24, 48, 96, 168, 336, and 672 h, during which thymol vapor was released from the SiO_2_ MCs. After each exposure time, those SiO_2_ MCs containing the encapsulated thymol were inserted into centrifuge tubes prefilled with 35 mL analytical grade ethanol and then centrifuged (8500 rpm, 30 min). Under these conditions, the SiO_2_ MCs containing the thymol break slightly so that the exposed thymol is dissolved in the ethanol. An additional centrifugation with fresh ethanol was applied to verify complete thymol extraction from the MCs. The thymol concentration (dissolved in ethanol) was calculated from the pre-calibrated curve equation (λ = 276 nm max). Measuring thymol contents was performed in triplicates.

#### 2.2.8. Durability Test of Oil-Filled Hydrophilic Silica Coatings onto Polypropylene Films

The durability of the coatings was evaluated by adhesion and abrasion tests. The test [24] was performed using SiC sandpaper with 240 grids in a row grinding surface. The coated films were placed against the glass paper, while a normal pressure of 1.96 kPa was applied to the sample and moved in one direction back and forth on the glass paper for 10 cm, both vertically and horizontally. This was performed a total of 50 times, both vertically and horizontally, for each coated sample. In order to verify the durability of the coating, FTIR was measured after every five repetitions. For the adhesion tests, an adhesive tape was firmly pressed onto the coated film and then slowly peeled off, as described in the literature [25]. The procedure was performed 25 times for each coated sample.

#### 2.2.9. Bioassay for Antiviral Activity against Tomato Brown Rugose Fruit Virus (ToBRFV)

The antiviral activity of hydrogen peroxide-filled SiO_2_ MCs against ToBRFV was evaluated using a bioassay on *Nicotiana glutinosa* plants. Varying weights of HP-filled SiO_2_ MCs (5 mg and 100 mg) were placed in small porous bags within Petri dishes. A 1 mL aliquot of 1:5000 diluted ToBRFV solution was applied to the side of each bag and incubated for 24 h at room temperature. Subsequently, 0.2 mL of phosphate buffer was added to extract any remaining viral particles. *N. glutinosa* leaves were prepared by spraying with silicon carbide (Carborundum) to facilitate viral entry. The leaves were then exposed to the collected liquid from the Petri dishes. Local lesions (LLs), indicating viral infection, were counted 4 days post-inoculation. Each treatment was replicated on three leaves from each of the three plants, totaling nine leaves per treatment. The positive control consisted of *N. glutinosa* leaves sprayed with Carborundum and directly exposed to a 1:5000 dilution of ToBRFV solution with 0.2 mL of phosphate buffer. A negative control using empty SiO_2_ MCs (without entrapped HP) was also included. The reduction in LL count compared to controls was used as an indicator of antiviral activity, with fewer LLs suggesting a decrease in viral load due to the treatment.

#### 2.2.10. Evaluation of Anti-Mold Properties of Thymol-Filled SiO_2_ MCs Coatings

The effectiveness of P(NN’BTMSPU)/thymol-filled SiO_2_ MCs coatings against mold growth was evaluated under favorable conditions for mold development. Petri dishes were prepared with 4 g of water and 3 g of hay to create a moist environment. PP/P(NN’BTMSPU)/thymol films containing SiO_2_ MCs were placed on top of the hay and covered with a Petri dish lid. Plates were incubated at room temperature with increased humidity for 21 days. Mold growth was monitored daily by measuring the area of mold growth with a ruler and calculating its percentage relative to the total plate area. Two independent replicates were performed for each treatment. The positive control included hay samples without any coating, while the negative controls used PP films without thymol-filled SiO_2_ MCs.

### 2.3. Characterization of the SiO_2_ Micro/Nano-Particles and PP Films

#### 2.3.1. Environmental Scanning Electron Microscope (E-SEM)

For morphological characterization, distribution, and size measurements of the average particle size distribution and the various coatings on top of the polymeric films, SEM images were taken using SEM Model JSM-840 Tokyo, Japan, with magnifications of 500, 2000, 10,000, 60,000, and 200,000. Dry-coated polymeric films and dry powder of SiO_2_ MCs were attached to a silicon wafer with carbon tape and coated with iridium prior to the E-SEM examination.

#### 2.3.2. Transmission Electron Microscopy (TEM)

The dry diameter and morphology of the SiO_2_ M/NPs were investigated using TEM (JEOL 1400, Jeol, Ltd., Tokyo, Japan). A small volume of M/NPs suspension was diluted with ethanol and dried on a copper grid. The grid was then examined by TEM.

#### 2.3.3. Fourier Transform Infrared Spectroscopy/Attenuated Total Reflectance (FTIR/ATR)

FTIR/ATR measurements of non-coated and coated PP films and noncoated corona-treated PP were performed using a Bruker, Billerica, MA, USA, ALPHA-FTIR QuickSnap Sampling Module equipped with a platinum FTIR diamond module Ddcd to examine functional groups of the different films.

#### 2.3.4. Atomic Force Microscopy (AFM)

The surface topography and roughness of the different films were measured using Rq roughness average values. AFM measurements were performed with a Bio Fast Scan Scanning Probe Microscope (Bruker AXS, Billerica, MA, USA). All images were obtained using the Bruker Peak Force QNM (PeakForce™ quantitative nanomechanical mapping).

#### 2.3.5. Brunauer–Emmet–Teller (BET)

The surface area of the MCs was measured using the Brunauer–Emmet–Teller (BET) method. The surface area and average pore size were determined using a NOVA 3200E analyzer from Quantachrome Instruments, Boynton Beach, FL, USA. Samples were degassed at 120 °C for 120 min to remove adsorbed gases. Nitrogen adsorption isotherms were then measured at liquid nitrogen temperature (−196 °C).

## 3. Results

**Figure 2:**
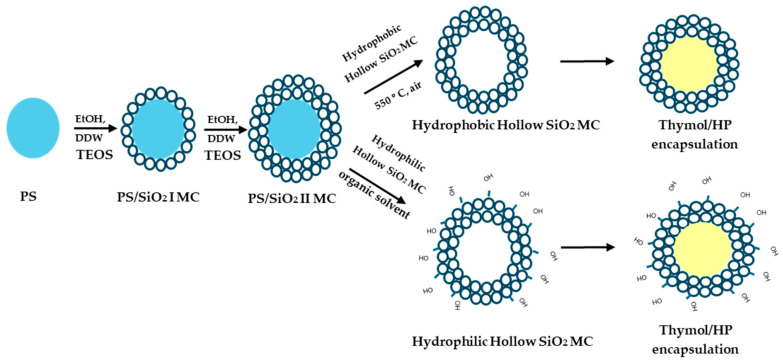
General scheme describing the synthesis of porous hydrophobic and hydrophilic SiO_2_ MCs.

Figure 2 illustrates the general synthesis presented in the article on porous hydrophobic and hydrophilic SiO_2_ MCs. PS microparticles of a narrow size distribution were produced by the dispersion polymerization mechanism of styrene. Uniform free core–shell PS/SiO_2_ microparticles were produced by seeded polymerization of Si(OEt)_4_ onto the PS microparticles dispersed in an ethanol/water continuous phase. The formed core–shell PS/SiO_2_ microparticles are composed of sintered SiO_2_ M/NPs, 36–200 nm diameter, coated onto the PS core particles. The diameter and thickness of the coated SiO_2_ M/NPs were controlled by changing the polymerization parameters of the Si(OEt)_4_ monomers, e.g., monomer concentration, polymerization pH, solvent, etc. Hydrophobic porous SiO_2_ MCs (to be used in the dry state) were produced by burning off in regular atmospheric conditions the dry PS/SiO_2_ MCs at 500 °C. Because of this high temperature, the core PS decomposed to CO_2_ and H_2_O vapors and the silanol groups (Si-OH), particularly surface silanol groups, to siloxane (Si-O-Si) and H_2_O vapors. Hydrophilic porous hollow SiO_2_ MCs (to be used in a dry state or in aqueous solutions) were produced via dissolution of the PS core of the PS/SiO_2_ particles with an appropriate organic solvent such as acetone, thereby preserving the silanol concentration during the removal of the PS core from the core–shell particles.

### 3.1. Polystyrene Core Microspheres

**Figure 3:**
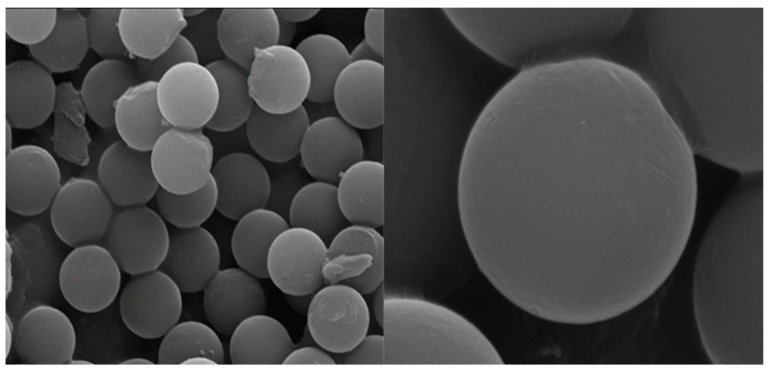
*E*-SEM photomicrographs of the PS microparticle surfaces.

Narrow-size PS microspheres of 2.7 ± 0.15 μm diameter and a smooth surface were produced as described in Section 2.2.1, see Figure 3.

### 3.2. Hydrophobic Silica Microcapsules

#### 3.2.1. Multistep Synthesis and Characterization of Hydrophobic Silica Microcapsules

In this study, we found that properties of the SiO_2_ shell, including its thickness and particle size distribution, can be fine-tuned by adjusting various parameters of the TEOS polymerization process. These parameters include TEOS concentration, ethanol-to-water ratio, and pH of the reaction medium. Table 1 provides a comprehensive overview of the different conditions we investigated to optimize the formation of SiO_2_ M/NPs on the PS core, resulting in well-defined PS/SiO_2_ core/shell structures.

**Figure 4:**
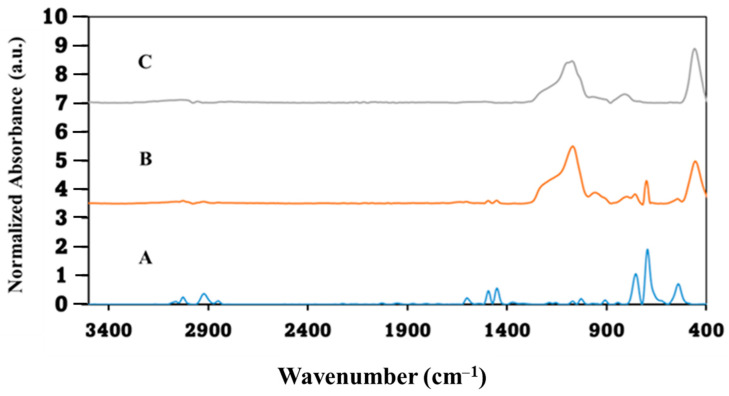
FTIR/ATR of: (**A**) PS core microparticles, (**B**) PS/SiO_2_ core–shell microparticles, and (**C**) SiO_2_ MCs.

In a subsequent stage, the polystyrene template microspheres were coated with varying sizes of SiO_2_ M/NPs. To confirm the presence of distinct functional groups at each synthesis step, FTIR/ATR analysis was performed. Spectrum 4A shows clear peaks at 700 and 750 cm^−1^ corresponding to the aromatic ring and peaks at 1452 and 1495 cm^−1^ corresponding to C=C bonds, indicating the presence of aromatic PS rings and confirming the presence of PS. Furthermore, the spectral profile of sample 2, shown in spectrum 4B, confirms the presence of SiO_2_ particles on the core PS structures. This is evident by absorption bands at 1080 and 1230 cm^−1^, which are attributed to Si-O-Si bridge stretching vibrations and Si-OH bonds. After the removal of the PS templates, a decline in the aromatic PS peaks is observed, indicating the formation of SiO_2_ MCs, as illustrated in Figure 4C.

**Figure 5:**
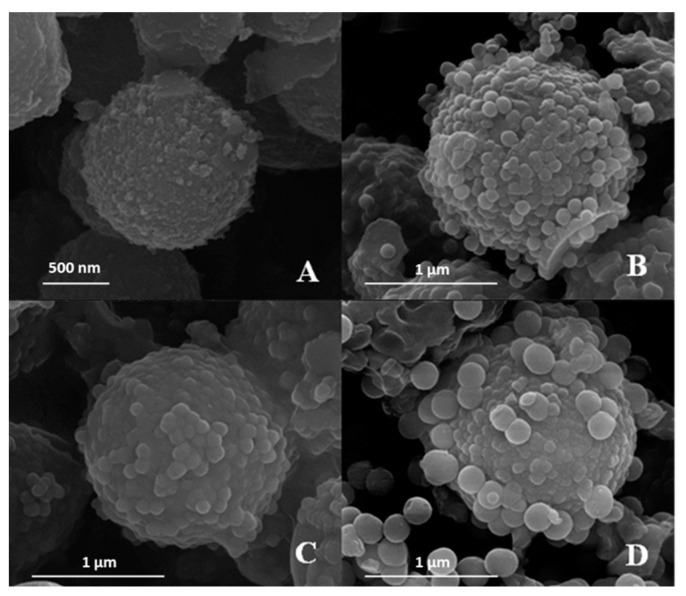
E-SEM images: (**A**) PS microspheres coated with SiO_2_ M/NPs 36 ± 5.8 nm. (**B**) PS microspheres coated with SiO_2_ M/NPs 103.9 ± 2.4 nm. (**C**) PS microspheres coated with SiO_2_ M/NPs 130.2 ± 2.8 nm. (**D**) PS microspheres coated with SiO_2_ M/NPs 200.8 ± 3.1 nm.

E-SEM analysis was carried out to examine the surface morphology and microstructure of PS/SiO_2_ core–shell particles. Figure 5 provides E-SEM images of uncoated PS surfaces, illustrating their smooth and featureless nature. In contrast, the in situ Stöber polymerization-mediated coating of SiO_2_ M/NPs resulted in a uniform layer of SiO_2_ particles with well-defined dimensions, enveloping the PS cores. This process produced randomly distributed core–shell-like particles, as depicted in Figure 5A–D.

As mentioned earlier, the presence of hierarchical surface structures on both micro and nano scales is a key characteristic of naturally porous particles. This hierarchical architecture consists of PS cores encased in SiO_2_ particles of various sizes, ranging from 36 ± 5.8 nm to 200.8 ± 3.1 nm (refer to Table 1). Due to their distinct properties, these microcapsules are suitable for a wide range of applications.

**Figure 6:**
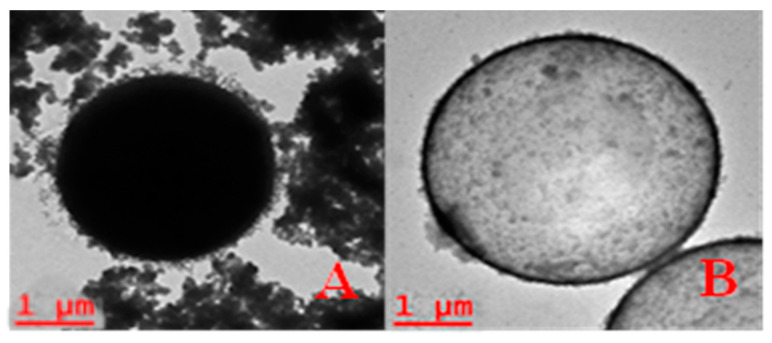
TEM images: (**A**) PS microspheres coated with SiO_2_, (**B**) hydrophobic SiO_2_ MC.

After successfully preparing the PS/SiO_2_ core–shell particles, a heat treatment at 550 °C in the presence of air was applied overnight to induce the combustion and decomposition of the PS core, resulting in the formation of hydrophobic SiO_2_ MCs. This process was validated through TEM characterization, as shown in Figure 6.

The hydrophobic SiO_2_ MCs float on water due to their hydrophobic surface characteristics. To fill them with aqueous solutions, it is essential to use a vacuum process, as described in Section 2.2.4.

#### 3.2.2. Surface Area of Silica Microcapsules

The surface area in chemistry holds significant implications, directly impacting a wide range of chemical processes and interactions. Furthermore, the surface area plays a crucial role in determining material properties, including aspects such as absorbency, mechanical strength, and thermal flexibility. Understanding and controlling surface area becomes essential for optimizing chemical reactions, developing efficient catalysts, and tailoring material properties to suit a diverse array of practical applications. To estimate the surface area, a Brunauer–Emmet–Teller (BET) instrument was used, involving two separate series of SiO_2_ MC tests. The initial series focused on examining the gaps between SiO_2_ MCs while maintaining identical SiO_2_ layer amounts (four layers) but varying the dimensions of the silica M/NPs coating. In a subsequent series, the focus shifted to SiO_2_ MCs with identical SiO_2_ particle dimensions (130 ± 2.8 nm) but varying layer numbers. Below is a summary of the experimental results, which shed light on the effect of these parameters on the surface area of the SiO2 MCs (Table 2).

The tabulated data reveal a clear trend, a reduction in SiO_2_ particle diameter leading to a significant increase in the surface area of the MCs, as expected. This effect is accompanied by a decrease in material porosity, causing the resulting surface area to fall below the critical threshold of 100 m^2^/g. In the subsequent series, a similar observation is noted: increasing the number of SiO₂ M/NPs layers on the polystyrene core resulted in a reduction in surface area, probably by blocking the pores. This trend confirms a decrease in material porosity and highlights the impact of the number of layers on the accessible surface area.

#### 3.2.3. Cumulative Release Rate of Hydrogen Peroxide Vapors from the Silica Microcapsules of Varied Silica Layers

The HP content in SiO_2_ MCs (130 ± 2.8 nm dry diameter) as a function of the number of silica layers was determined through a comparative analysis of the weights of the SiO_2_ MCs before and after being filled with HP solution. Initially, the weight of the empty MCs was measured, and then the weight of the MCs after being filled with the HP solution (6% wt HP in water) in a vacuum process was measured, as described in Section 2.2.4. The change in the particle weight before and after filling serves as an indication of the quantity of entrapped HP (6% wt) in the MCs. 

Table 3 presents the weight ratio obtained between HP and SiO_2_ MCs with varying layers. As indicated in this table, an increase in the number of layers correlates with a decrease in the quantity of entrapped HP characterized by a reduced surface area. Specifically, for SiO_2_ MCs with 2, 6, and 10 layers, the weight ratio of [HP]/[SiO_2_] diminished from 0.32 to 0.15 and 0.06, respectively. It is conceivable that an extensive presence of coating layers obstructs the hollow cavities, posing a challenge for the HP solution to permeate.

To investigate the rate of HP release from the various SiO_2_ MCs, the SiO_2_ MCs saturated with HP underwent a titration process that included the use of a pre-calibrated KMnO_4_ after different time intervals (0–168 h), as previously described [17,18]. The number of moles of HP within these MCs was then calculated and described in Table 4. As observed in Table 4, SiO_2_ MCs with two layers exhibited the slowest release rate of HP compared to SiO_2_ MCs with a higher number of layers. For instance, after one week, 41% of the HP moles remained in SiO_2_ microcapsules with two layers, whereas 35% and 16% were retained in SiO_2_ MCs with 6 and 10 layers, respectively.

It is possible that there is a correlation between the large surface area of SiO_2_ MCs with few layers and the release rate of the HP. MCs with few layers have a large surface area that allows for binding and interaction between the HP molecules in the inner part of the microcapsules. Therefore, the HP release rate from these SiO_2_ microcapsules is slower. In other words, SiO_2_ microcapsules with a reduced number of layers demonstrated greater effectiveness in the controlled release of HP, attributed to their increased surface area.

In summary, reducing the number of layers in the SiO_2_ microcapsules enhances both the penetration of HP into the particles and increases the effectiveness in the controlled release of HP from the SiO_2_ microcapsules. Additional advantages associated with SiO_2_ microcapsules featuring a low number of coatings are the cost reduction and acceleration of their preparation process. These advantageous properties, especially the high containment capacity and controlled release, make these particles promising materials for potential applications in the agricultural industry.

#### 3.2.4. Effect of Hydrogen Peroxide Vapors on the ToBRFV Virus

**Figure 7:**
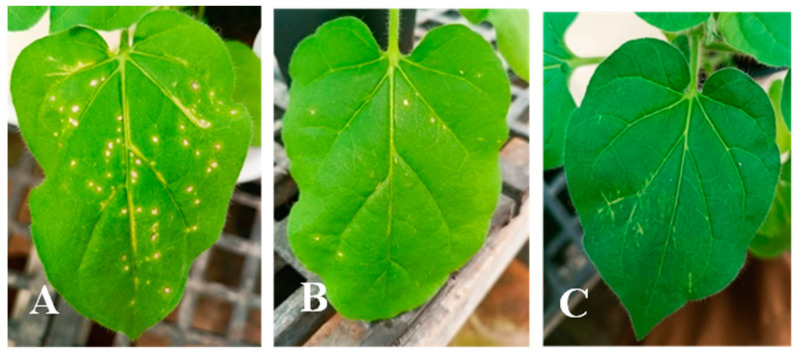
Biological assay of ToBRFV on *Nicotiana tabacum* of the SiO_2_ MC. Virus-infected seedlings exposed to (**A**) control SiO_2_ MC, (**B**,**C**) 5 mg, and 100 mg of HP-filled SiO_2_ MC, respectively. (**A**) Empty SiO_2_ microcapsules (control), (**B**) HP-filled SiO_2_ microcapsules (5 mg), and (**C**) HP-filled SiO_2_ microcapsules (100 mg).

We aimed to evaluate the effectiveness of HP-filled SiO_2_ MCs against the spread of the tomato brown rugose fruit virus (ToBRFV). We conducted tests on tobacco plants, using an indirect contact diffusion method where HP vapors were released from the HP-filled SiO_2_ MCs (100 mg, HP 6%) packed in empty silica gel packages to the gas phase containing the virus. The bioassay involved quantifying local lesions (LL) on tobacco plant leaves with the virus being exposed to HP-filled SiO_2_ MCs before the biological assessment. The results are summarized and demonstrated in Table 5 and Figure 7.

The results presented in Table 5 and Figure 7 demonstrate a nearly complete virucidal effect when the virus was exposed to the HP vapors of HP-filled SiO_2_ MCs through indirect contact. In contrast, the control SiO_2_ MCs had no impact on virus viability. Control tobacco seedlings (Figure 7A) exhibited extensive proliferation of LLs, while virus-infected seedlings exposed to SiO_2_ MCs filled with HP (Figure 7B,C) displayed healthy leaves with no signs of LLs. These findings suggest the promising antiviral potential of HP-filled SiO_2_ MC in combatting the spread of ToBRFV.

### 3.3. Hydrophilic Silica Microcapsules

#### 3.3.1. Synthesis of Hydrophilic Silica Microcapsules

**Figure 8:**
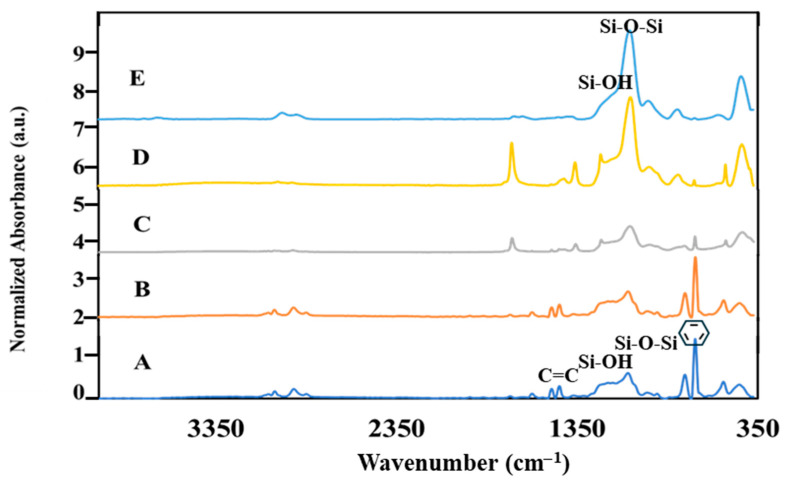
Monitoring PS dissolution using FTIR/ATR: (**A**) PS/SiO_2_ microparticles. (**B**–**E**) PS/SiO_2_ microparticles after one, two, three, and four rinses with acetone, respectively.

Hydrophilic SiO_2_ MCs were prepared and characterized as described in Section 2.2.3. FTIR/ATR spectra shown in Figure 8 illustrate the change caused by a few washings of the PS/SiO_2_ MC with acetone. As noted, PS exhibits distinct peaks in the spectrum, and the reduction in their intensity correlates with the rate of PS dissolution. These peaks are located at 700 and 750 cm^−1^ for aromatic rings and at 1452 and 1495 cm^−1^ for C=C bonds. Notably, after undergoing four washes with acetone, the characteristic PS peaks have completely disappeared, indicating the thorough hollowing of the particles to obtain hydrophilic SiO_2_ MCs.

**Figure 9:**
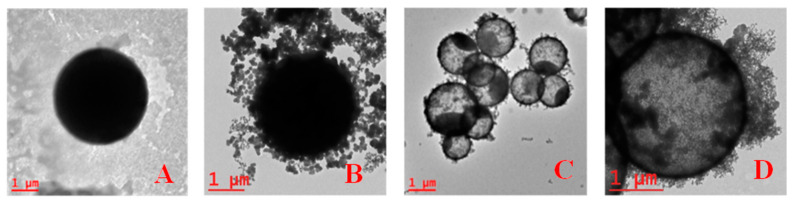
TEM images: (**A**) PS microspheres, (**B**) PS/SiO_2_ core–shell microparticles, (**C**) half-empty SiO_2_ MC, and (**D**) hydrophilic SiO_2_ MC.

The progression of this process is also evident through TEM. Figure 9 provides a comparative analysis showing three stages: PS microsphere, PS/SiO_2_ core–shell particles, and particles in an intermediate and last stage of PS dissolution in acetone. Notably, these particles exhibit an empty structure, albeit with residual PS remnants, indicating a gradual hollowing process. This observation underscores the successful formation of hydrophilic SiO_2_ MCs during the synthesis.

During this process of PS core dissolution, we kept the silanol concentration stable, thus maintaining the hydrophilic property of the resulting SiO_2_ MCs.

#### 3.3.2. Release Kinetics of Thymol from Silica Microcapsules

**Figure 10:**
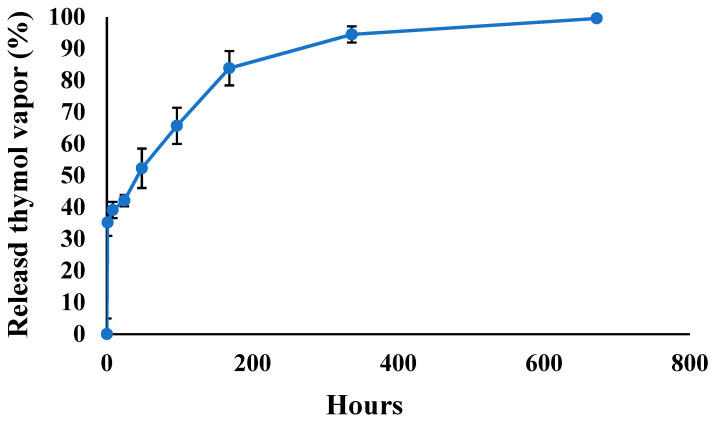
Release profile of thymol vapor from SiO_2_ MCs over four weeks.

To evaluate the release kinetics of thymol vapor from the SiO_2_ MCs, we conducted a time-dependent study of the cumulative thymol release. The release kinetics of thymol vapor (over a period of 672 h, four weeks) from the SiO_2_ MCs was calculated by reduction of the thymol content in the SiO_2_ MCs at time 0 from the content remaining in the MCs at each time period. Figure 10 illustrates the cumulative release profile of thymol vapor over time.

The release profile shows an initial rapid release of thymol within the first 24 h, during which approximately 40% of the encapsulated thymol was released from the SiO_2_ MCs. This was followed by a more gradual release over the subsequent days. By 96 h, about 80% of the initial thymol content had been released, with the release rate slowing down thereafter. At the end of the 168 h period (one week), approximately 87% of the original thymol content had been released from the microcapsules. The release continued at a slower rate over the following weeks, with about 92% released after two weeks (336 h) and reaching 95% after four weeks (672 h).

This release pattern suggests that the SiO_2_ MCs provide an initial burst release of thymol, which could be beneficial for immediate antimicrobial action. This is followed by a sustained release that could maintain the antimicrobial effect over an extended period. Most of the thymol (about 87%) is released within the first week, providing a strong initial antimicrobial effect. The slower release over the subsequent weeks could help maintain a lower level of antimicrobial activity for up to a month.

The sustained release characteristics of these SiO_2_ MCs make them promising candidates for long-term antimicrobial applications in agricultural settings, such as the hay preservation demonstrated in the present study. The initial high-release rate could quickly establish an antimicrobial environment, while the extended-release over several weeks could help maintain protection against mold growth during storage or transportation of agricultural products.

#### 3.3.3. Oil-Filled Hydrophilic Silica Coatings on PP Films

Oil-filled SiO_2_ MC coatings on PP films were prepared by using a modified Stöber polymerization procedure of the monomeric bipodal silane compound N,N′-bis (3-trimethoxysilylpropyl) urea, (NN’BTMSPU, see Figure 1) in the absence or presence of thymol-filled SiO_2_ MC in EtOH/H_2_O continuous phase, as described in Section 2.2.5. The solution was shaken at room temperature for 2–3 min then spread on the oxidized corona-treated PP film with a Mayer rod. The bipodal silane was used for improving the grafting process to the corona-treated PP surface, thereby improving the coating stability.

**Figure 11:**
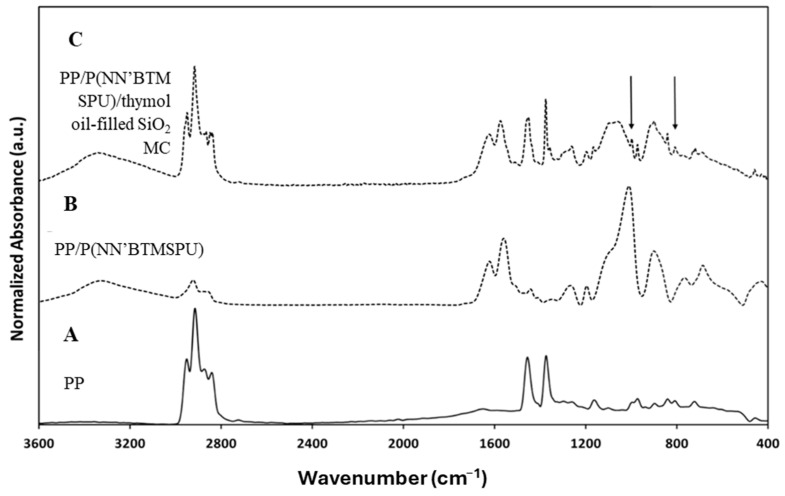
FTIR spectra of (**A**) oxidized PP film, (**B**) PP/P(NN’BTMSPU) film, and (**C**) PP/P(NN’BTMSPU)/thymol oil-filled SiO_2_ MC film.

Figure 11 shows the FTIR/ATR spectra of corona-treated PP, PP/P(NN’BTMSPU), and PP/P(NN’BTMSPU)/thymol-filled hydrophilic SiO_2_ MC films.

The characteristic peaks of PP are at 808 cm^−1^ (C-C stretching), 840 cm^−1^ (C-H stretching), 973, 998 and 1166 cm^−1^ (CH_3_ rocking), and 1376 and 1456 cm^−1^ (CH_3_ symmetrical bending).

The characteristic absorption peaks at 1132 cm^−1^ corresponding to the Si-O-Si stretching band appear for both PP/P(NN’BTMSPU) and PP/P(NN’BTMSPU)/thymol-filled SiO_2_ MC films. In particular, the peak at 1041 cm^−1^ is related to the absorbance of Si-OH. The peak at 1657 cm^−1^ is assigned to the imidic C=O stretching band (N-C=O). The main peaks of thymol ring vibration are seen at 806 cm^−1^ and 1253 cm^−1^.

**Figure 12:**
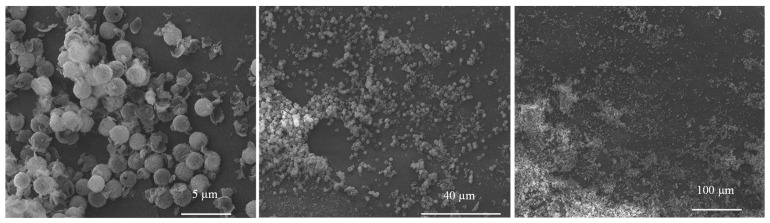
SEM images of PP/P(NN’BTMSPU)/thymol oil-filled SiO_2_ MC film under different magnifications.

Thin-coated PP films containing SiO_2_ MCs were characterized using SEM (Figure 12). A relatively uniform dispersion of oil-filled SiO_2_ MCs can be observed at different magnifications, showing the PP film’s thymol release activity.

**Figure 13:**
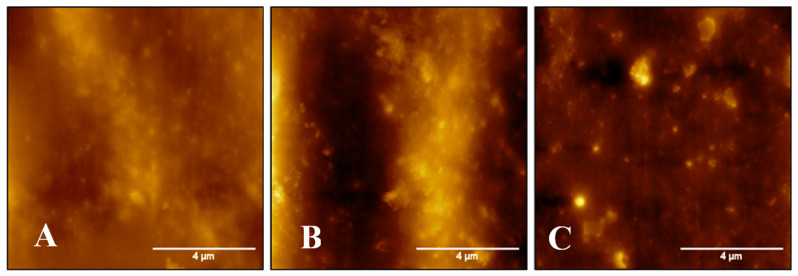
AFM spectra of (**A**) oxidized PP film, (**B**) PP/P(NN’BTMSPU) film, and (**C**) PP/P(NN’BTMSPU)/thymol-filled SiO_2_ MC film.

Table 6 and Figure 13 illustrate that the oxidized PP, PP/P(NN’BTMSPU), and PP/P(NN’BTMSPU)/thymol oil-filled SiO_2_ MC films possess varying degrees of surface roughness. The PP and PP/P(NN’BTMSPU) films showed relatively similar levels of roughness, 22.2 and 25.2 nm, respectively. However, the PP/P(NN’BTMSPU)/thymol-filled SiO_2_ MC film showed a significant increase in roughness (44.5 nm) as compared to the other films.

#### 3.3.4. Anti-Mold Efficacy of Thymol-Filled SiO_2_ MCs Coatings

**Figure 14:**
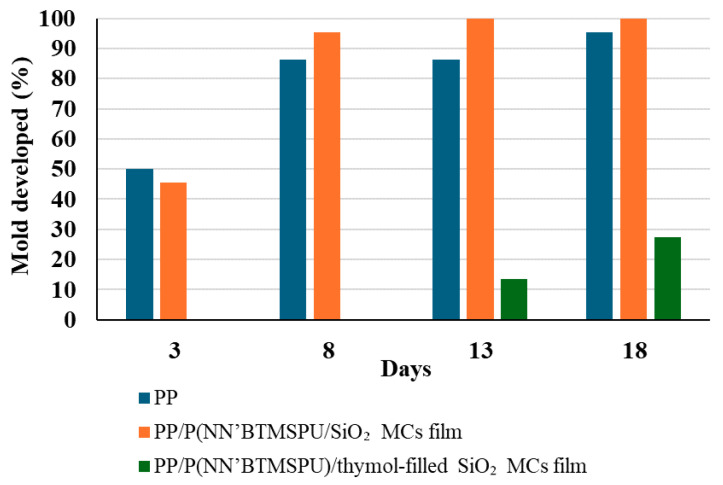
A quantitative comparison of mold coverage.

**Figure 15:**
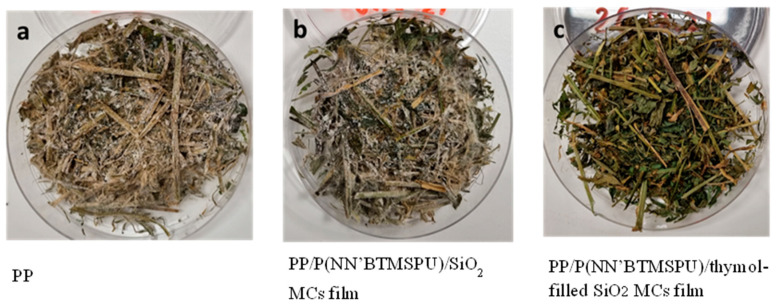
Images of PP (**a**), PP/P(NN’BTMSPU/SiO_2_ MCs film (**b**), and PP/P(NN’BTMSPU)/thymol-filled SiO_2_ MCs film (**c**) after two weeks.

The anti-mold properties of PP/P(NN’BTMSPU)/thymol-filled SiO_2_ MC coatings were evaluated over a period of 21 days. As shown in Figure 14, after two weeks of the experiment, only the PP/P(NN’BTMSPU)/thymol coatings containing SiO_2_ MCs remained free of visible mold growth in both replicates. In contrast, control samples without thymol became moldy after 2 days. In one of the replications, slight mold growth was observed in the thymol-containing coating after 13 days, but this growth was significantly lower than in the control samples. Figure 14 provides a quantitative comparison of mold coverage percentages for different coatings after 21 days, clearly illustrating the superior anti-mold performance of the thymol-containing coating. These findings demonstrate the strong mold-inhibiting properties of thymol-containing SiO_2_ coatings, with effective protection lasting at least two weeks under very favorable conditions for mold growth. The results shown in Figure 15 represent one of the two experimental replicates.

The durability of the thymol-filled SiO_2_ MC coatings was evaluated using an adhesion tape and abrasion tests (see Section 2.2.8), and afterward, the films underwent characterization by FTIR. All coatings exhibited the same peaks as shown before the tests (Figure 8), indicating that the coatings are stable and resistant to mechanical abrasions. It should also be noted that the coating remained stable after at least two months at room temperature conditions. This indicates their long-term durability and suitability for various applications.

## 4. Discussion

The choice of using hydrophobic or hydrophilic SiO_2_ MCs is dependent on the desired application and release mechanism. Hydrophobic SiO_2_ MCs were selected for HP vapor release to allow controlled vapor release under dry conditions. Otherwise, water and HP dissolved in water may also release from the entrapped 6% HP aqueous solution. In other words, the hydrophobic nature of these microcapsules helps prevent premature release of the HP in humid environments, ensuring it is available for vapor-phase antiviral action when needed. In contrast, hydrophilic SiO_2_ MCs were chosen for thymol encapsulation to improve the dispersion of the SiO_2_ MCs in the hydrophilic N,N′-Bis(3-Trimethoxysilylpropyl)urea (NN’BTMSPU) coating on corona-treated PP film, and to prevent leaching of liquid thymol through hydrophobic SiO_2_ MCs systems and promotes thymol vapor release only.

The hollow silica microcapsules synthesized and characterized in this study demonstrate promising potential for agricultural applications, particularly for controlled release of active ingredients and improved crop protection. The ability to tune properties such as shell thickness, porosity, and surface functionality allows for the customization of release kinetics and targeting.

Compared to other hollow microcapsule systems reported in the literature, the polystyrene templating method used here offers good control over particle size and morphology. For example, Yoon et al. [26] produced hollow silica microspheres using a soft templating emulsion method, but with much higher polydispersity (CV of 50.5%) compared to the monodispersed capsules used in the current work. The hard templating approach allows for more precise control over the internal cavity size.

The shell thickness range of 10–150 nm achieved here is comparable to other reports using polystyrene templates [27,28,29]. However, the ability to produce larger micron-sized capsules addresses a gap in the literature, as most previous work focused on sub-micron particles. The larger size may be advantageous for a higher loading capacity of active ingredients.

In terms of agricultural applications, the hydrogen peroxide-loaded capsules demonstrated here for virus inactivation show similar efficacy to other encapsulated peroxide systems. For example, Malka et al. [30] reported successful mold inhibition using hydrogen peroxide incorporated into PVA/PVP hydrogels. The silica shell approach provides an alternative delivery vehicle that may offer improved stability and controlled release compared to polymer matrices.

The use of thymol as an antifungal agent aligns with growing interest in essential oil-based crop protection [31]. The silica encapsulation strategy demonstrated here could help overcome volatility and stability issues that have limited widespread adoption of essential oils in agriculture. The thin coatings produced on polypropylene films also showcase the versatility of the microcapsules for creating functional surfaces.

Compared to other reported agricultural delivery systems, key advantages of the hollow silica microcapsules include their high loading capacity, controllable porosity for tuning release rates, and ability to protect sensitive active ingredients. The inorganic silica shell also provides good thermal and chemical stability. However, further work is still needed to optimize release kinetics and evaluate long-term efficacy under field conditions.

The release profile of thymol from the SiO_2_ MCs, with approximately 87% cumulative release over 168 h, demonstrates the potential for these microcapsules to provide sustained antimicrobial activity. This is particularly relevant for applications like hay preservation, where long-term protection against mold growth is crucial. The initial burst release observed in the first 24 h could provide rapid antimicrobial action, while the subsequent slower release could maintain protection over an extended period.

Overall, this study advances the development of hollow silica microcapsules as a promising platform technology for next-generation agrochemical delivery. The ability to precisely engineer particle properties at the nano/micro-scale opens new possibilities for improving the efficiency and environmental profile of crop protection products. Future work should focus on scaling up production, expanding the range of encapsulated active ingredients, and conducting extensive field trials to validate performance.

## 5. Summary and Future Study

The present study has advanced our understanding of SiO_2_ MCs synthesis and applications, particularly in the agricultural sector. The innovative approach of creating both hydrophobic and hydrophilic SiO_2_ MCs with controlled surface properties and release kinetics represents a significant step forward in materials science. Notably, the HP-filled SiO_2_ MCs demonstrated remarkable virucidal properties against the ToBRFV, showcasing their potential as a novel antiviral agent in crop protection. Additionally, the thymol-filled SiO_2_ MCs incorporated into polymer films exhibited promising anti-mold properties, offering a sustainable solution for hay preservation. These findings open new avenues for the development of environmentally friendly and efficient agricultural technologies. Hydrophobic SiO_2_ MCs containing aqueous solution can be dispersed in organic solvents while storing the water within the capsules. Hydrophilic SiO_2_ MC containing organic solutions can also disperse nicely in water while keeping the organic content within the capsules. Future research should focus on optimizing the release profiles of active ingredients from these MCs, exploring their long-term stability under various environmental conditions, and investigating their efficacy against a broader spectrum of plant pathogens and pests. Furthermore, scaling up the production process and conducting field trials are crucial steps towards the practical implementation of these innovative materials in agricultural settings.

## Figures and Tables

**Table 1:** Synthetic parameters are used to form different sizes of SiO_2_ M/NPs.

SiO_2_ M/NPs Size (nm)	Ethanol (mL)	Water (mL)	NH_4_OH (28%) (mL)	TEOS (mL)
36.9 ± 5.8	23.5	0.4	0.35	0.8
103.9 ± 2.4	23.5	0.4	1.0	0.8
130 ± 2.8	19.0	1.0	0.67	1.5
200.8 ± 3.1	19.0	4.2	0.45	3.0

**Table 2:** SiO_2_ microcapsules surface area measurements using BET.

**First series-identical SiO_2_ layer amounts (four layers)**	
**Silica M/NPs diameter** **(nm)**	**Surface area** **(m^2^/g)**
36.9 ± 5.8	207.2 ± 16.7
103.9 ± 2.4	151.1 ± 23.3
130 ± 2.8	104.7 ± 33.6
200.8 ± 3.1	74.2 ± 32.7
**Second series-identical SiO_2_ particle dimensions (130 ± 2.8 nm), but varying numbers of layers**	
**No. of SiO_2_ M/NPs layers of the MC**	**Surface area** **(m^2^/g)**
1	252.3 ± 14.0
4	104.7 ± 33.6
10	72.0 ± 47.6

**Table 3:** Comparison of [HP]/[SiO_2_] for the different series containing a variable number of SiO_2_ layers.

No. of SiO_2_ Layers	[HP]/[SiO_2_] (*w*/*w*)
2	0.31
6	0.15
10	0.06

**Table 4:** Tracking the rate of HP release from SiO_2_ MCs over time.

	HP (Mole) Entrapped in the SiO_2_ MCs (130 ± 2.8 nm)						
Layers	0	1 h	8 h	24 h	48 h	96 h	168 h
2	3.9	3.4	3.2	2.9	1.9	2.2	1.6
6	3.1	2.4	2.1	1.8	1.5	1.3	1.1
10	2.4	1.8	1.6	1.5	1.3	0.9	0.4

**Table 5:** ToBRFV bioassay of infected tobacco seedlings.

Sample	Seedling No.	LLs	Sample	Seedling No	LLs
Positive control	1	48	HP-filled SiO_2_ MC (100 mg)	1	1
Positive control	2	76	HP-filled SiO_2_ MC (100 mg)	2	2
Positive control	3	37	HP-filled SiO2 MC (100 mg)	3	0
Positive control	4	66	HP-filled SiO_2_ MC (100 mg)	4	0
Empty SiO_2_ MC	1	74	HP-filled SiO_2_ MC (5 mg)	1	7
Empty SiO_2_ MC	2	49	HP-filled SiO_2_ MC (5 mg)	2	5
Empty SiO_2_ MC	3	63	HP-filled SiO_2_ MC (5 mg)	3	1
Empty SiO_2_ MC	4	41	HP-filled SiO_2_ MC (5 mg)	4	1

**Table 6:** Measured roughness (Rq) of oxidized PP film, (b) PP/P(NN’BTMSPU) film, and (c) PP/P(NN’BTMSPU)/thymol-filled SiO_2_ MCs film.

Sample	PP *	PP/P(NN’BTMSPU)	PP/P(NN’BTMSPU)/Thymol-Filled SiO_2_ MC
**Rq (nm)**	22.2 ± 5.15	25.2 ± 10.1	44.5 ± 1.5

* Corona-treated PP film at 400 W·min/m^2^.

## Data Availability

The original contributions presented in the study are included in the article, further inquiries can be directed to the corresponding author.
